# Breast cancer patient-derived explant cultures recapitulate *in vivo* drug responses

**DOI:** 10.3389/fonc.2023.1040665

**Published:** 2023-02-22

**Authors:** Solveig Pettersen, Geir Frode Øy, Eivind Valen Egeland, Siri Juell, Olav Engebråten, Gunhild Mari Mælandsmo, Lina Prasmickaite

**Affiliations:** ^1^ Department of Tumor Biology, Radium Hospital, Oslo University Hospital, Oslo, Norway; ^2^ Department of Oncology, Oslo University Hospital, Oslo, Norway; ^3^ Insitute for Clinical Medicine, University of Oslo, Oslo, Norway; ^4^ Department of Medical Biology, Faculty of Health Sciences, University of Tromsø/the Arctic University of Norway, Tromsø, Norway

**Keywords:** breast cancer, patient-derived xenografts, *ex vivo* cultures, organoids, explants, drug sensitivity

## Abstract

Assessment of drug sensitivity in tumor tissue *ex vivo* may significantly contribute to functional diagnostics to guide personalized treatment of cancer. Tumor organoid- and explant-cultures have become attractive tools towards this goal, although culturing conditions for breast cancer (BC) tissue have been among the most challenging to develop. Validation of possibilities to detect concordant responses in individual tumors and their respective cultures *ex vivo* is still needed. Here we employed BC patient-derived xenografts (PDXs) with distinct drug sensitivity, to evaluate different conditions for tissue dissociation, culturing and monitoring of treatment efficacy *ex vivo*, aiming to recapitulate the *in vivo* drug responses. The common challenge of discriminating between tumor and normal cells in the cultured tissue was also addressed. Following conventional enzymatic dissociation of BC tissue, the tumor cells stayed within the non-disrupted tissue fragments, while the single cells represented mostly normal host cells. By culturing such fragments as explants, viable tumor tissue could be maintained and treated *ex vivo*, providing representative indications on efficacy of the tested treatment. Thus, drug sensitivity profiles, including acquired chemoresistance seen in the PDXs, were recapitulated in the respective explants. To detect the concordant responses, however, the effect monitoring had to be harmonized with the characteristics of the cultured tissue. In conclusion, we present the feasibility of BC explants *ex vivo* to capture differences in drug sensitivity of individual tumors. The established protocols will aid in setting up an analogous platform for BC patient biopsies with the aim to facilitate functional precision medicine.

## Introduction

Patient-proximal models hold promise as a drug screening platform for personalized cancer therapy. Patient-derived xenografts (PDXs) in mice have long been considered among the most important models, although their use is limited due to low throughput, high costs and ethical issues (1, 2). More recently, cultures of patient-derived organoids (PDOs) and patient-derived explants (PDEs) have become attractive tools for assessing drug sensitivity *ex vivo* in a personalized manner (3, 4). The term “organoids” describes stem-cell derived self-organizing three-dimensional (3D) structures that recapitulate features of the tissue of origin and have the ability to be expanded *in vitro* for long-term (5, 6). PDOs of colorectal, pancreatic and prostate cancers were among the first successfully developed and employed for assessing drug sensitivity *ex vivo* (7–9). This, on the other hand, has been challenging for breast cancer (BC). Currently, PDOs for most cancer forms, also BC have been developed (3, 10–13). In contrast to PDOs, PDEs represent short-term cultures of small fragments of tumor tissue (4, 14). Since PDEs partially maintain the heterogeneity and the microenvironment of the tumor of origin, they provide an opportunity to explore drug responses within the authentic context (15, 16).

Access to patient biopsies, particularly throughout the course of treatment (i.e. at the start, when the tumors are sensitive, and later, when they develop resistance) is often limited. Thus, precious patient material is seldom available for testing experimental drugs, developing new assays or performing mechanistic studies on treatment response or resistance. Such studies still have to rely on model systems, and PDX-derived cultures (PDXCs) *ex vivo* are attractive alternatives. It has been demonstrated that PDXCs can predict responses to targeted drugs in the matching PDXs (10, 13). However, discrepancies in response between *in vivo* and *ex vivo* models have also been observed (17).

The protocols used for tumor tissue processing, culturing and read-out of drug sensitivity vary between different studies (10–13), suggesting that individual optimization might be needed. Here we aimed to establish conditions for evaluation of drug responses *ex vivo* by using tumor tissue from triple-negative breast cancer (TNBC) PDXs with distinct drug sensitivity. The goal was to recapitulate *ex vivo* the drug sensitivity profile of the parental PDXs.

## Materials and methods

### PDXs maintenance and treatment

**Figure 1:**
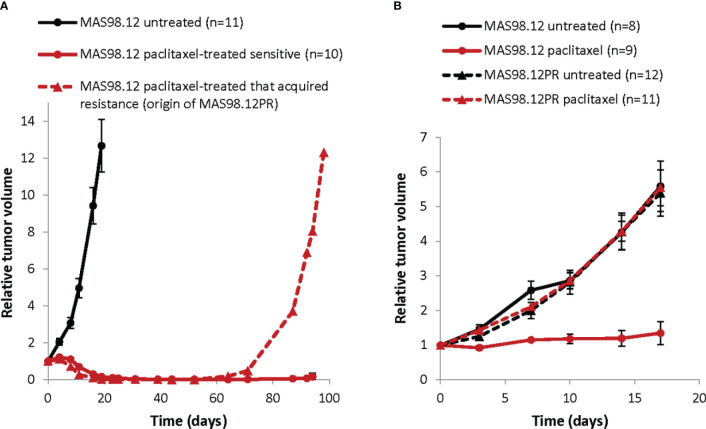
Growth of TNBC PDXs: paclitaxel sensitive MAS98.12 and the resistant sub-line MAS98.12PR with/without treatment. **(A)** Relative tumor volume (normalized to the volume at the day when the treatment was started) of the non-treated and the paclitaxel-treated (15 mg/kg, 2x/week for 3 weeks) MAS98.12. One of the treated tumors acquired resistance and re-grew, being the origin of the resistant sub-line MAS98.12PR; average ± SEM (n indicated in the legend). **(B)** Validation of the distinct sensitivity to paclitaxel (10 mg/kg, 2x/week) in the MAS98.12 and the daughter sub-line MAS98.12PR; average ± SEM (n indicated in the legend).

MAS98.12 PDX was established in-house and described previously (18). The paclitaxel resistant sub-line MAS98.12PR was established from a mouse bearing MAS98.12 tumor that was treated with 15 mg/kg paclitaxel twice per week for three weeks and after the initial response developed resistance as shown in **Figure 1A**. HBCx39 PDX was established at the Institute Curie (Paris, France) (19, 20) and was obtained through collaboration with Dr. Elisabetta Marangoni. All xenografts were maintained by serial passaging, implanting 1-3 mm^3^ pieces of the parental tumors into thoracic mammary glands of 6-8 week-old female HSD : Athymic Nude Foxn1nu mice locally bred at the Department of Comparative Medicine at the Norwegian Radium Hospital (Oslo, Norway). Before implantation, the mice were placed under anesthesia with sevoflurane (Baxter, Deerfield, IL, USA).

The treatments were initiated when tumor volume reached 60-200 mm^3^ and lasted for three weeks. Paclitaxel (Hospira UK Ltd, Hurley, UK or Sandoz, Basel, Switzerland) diluted in 0.9% saline was given intravenously (i.v), while capecitabine (Accord-UK, Barnstaple, UK) diluted in 40 mM citric buffer/5% gummi arabicum and everolimus (LC Laboratories, Woburn, MA, US) diluted in 0.5% methyl cellulose solution were given orally. Tumor growth was followed by measuring their size (length L and width W) using a caliper, and the tumor volume was calculated as: W^2^ x L x 0.5.

This study is compliant with all relevant ethical regulations regarding animal research and was conducted according to the recommendations of the European Laboratory Animals Science Association. All experiments involving animals were approved by the Norwegian Food Safety Authority (FOTS id 15499).

### PDX tissue dissociation and isolation of tissue fragments

Freshly resected or thawed cryopreserved (0.5 g tissue as 3-4 mm pieces/cryotube with 1 ml Recovery Cell Culture Freezing Medium (Gibco, Grand Island, NY, US)) tumors were minced with a scalpel and digested with 2 mg/ml collagenase IV and 100 μg/ml DNAse (both from Sigma-Aldrich, St.Louis, MO, USA) dissolved in advanced DMEM/F12 supplemented with Glutamax, HEPES and Penicilin/Streptomycin (concentrations/producers specified in **Supplementary Table S1**). The digestion was performed at 37°C on rotation for up to 1 h. Where indicated, additional mechanical dissociation using the gentleMACS dissociator (Miltenyi Biotec, Bergisch Gladbach, Germany) at “m-imp Tumor 03” settings were applied. The dissociated tissue suspension was diluted with phosphate-buffered saline (PBS)/1% bovine serum albumin (BSA) (both Sigma-Aldrich) and centrifuged at 18g for 4 min. The pellet was re-suspended and centrifuged again first at 32g, then at 200g for 4 min. The cell viability was monitored by staining aliquots with 0.2% trypan blue (NanoEntek, Seoul, Korea). Majority of dead cells remained in the supernatants, while the final pellet consists of a mixture of viable single cells and small non-disrupted tissue fragments. The final pellet was re-suspended in breast cancer organoid medium (OM) described by Sachs et al. (12) (specified in **Supplementary Table S1**).

Additional steps to remove normal mouse cells included plating re-suspended pellet in 24-well plates treated with anti-adherence Rinsing Solution (Stemcell Technologies, Cambridge, UK) and culturing in OM supplemented with 5 μM of the MDM2 inhibitor Nutlin-3 (Cayman, Ann Arbor, MI, USA), further called OM+. Nutlin-3 induces death in cells with wild-type TP53 i.e. normal cells, while tumor cells with lost/mutated TP53 stay viable (11). The PDXs used in this study harbor a mutation of the *TP53* gene (18, 19). Therefore, the tumor tissue can be subjected to Nutlin-3 selection for enrichment of cancer cells. Subsequently, the tissue suspension was filtered through a 100 µm cell strainer to collect fragments below this size that were further sedimented for 2-5 min. The resulting fragment-enriched pellet was used for establishment of PDXCs.

### PDXCs in Matrigel and treatment with drugs

The fragment-enriched pellet was resuspended in OM+ to a concentration of approximately 7 - 9 fragments/μl. Fragment counting was performed manually in a 10 μl droplet of suspension by using a light microscope. After addition of 30% Matrigel (Corning, New York, USA), a droplet of 10 µl containing approximately 50-60 fragments was added to each well in a 48-well plate, and the domes were allowed to solidify at 37°C for 30 min before addition of 190 µl of OM+. The next day, 200 µl of OM+ with the desired concentration of the drug was added. Half of the medium (+/- drug) was replaced twice per week.

### Scoring of treatment response in PDXCs

#### Analysis of fragment growth by measuring their total area

Each well was analyzed in real-time by using Incucyte^®^ S3 equipped with the organoid analysis software module (Sartorius, Gottingen, Germany). The module automatically detects fragment total area providing growth curves for control- and treated-explants.

#### Live/dead staining and calculation of a proportion of live cells in the fragments

The treated PDXCs and the respective untreated controls were stained with 2 μM calcein-AM (Sigma- Aldrich) for 30 min at 37°C followed by staining with 350 nM propidium iodide (PI) (Invitrogen, Waltham, MA, USA) for 30 min to distinguish live (green) and dead (red) cells, respectively. The stained cultures were analyzed by Olympus IX81 microscope equipped with a 4x objective and filters 488/527 (for calcein) and 540/590 (for PI) (Olympus, Tokyo, Japan). Three images per well together covering whole area of the dome were captured. Fiji/ImageJ (21), an open-source software for image processing was used to measure the calcein- and PI-signal area in pixels in each fragment. Proportion of live cells in the fragments was calculated in each well based on the equation “calcein-signal”/[“calcein-signal” + “PI-signal”].

#### Metabolic activity measurements by CellTiter-Glow assay

The PDXCs were prepared as above but in white 96-well plates with clear bottom (Corning, New York, NY, USA). After one week of treatment, CellTiter-Glo 3D reagent (Promega, Madison, WI, USA) was added at a ratio 1:2, and luminescence was measured by the Victor X3 plate reader (PerkinElmer, Waltham, MA, USA).

### Evaluation of the proliferative ability *ex vivo*

The proliferative ability of the dissociated PDX tissue *ex vivo* was evaluated by scoring EdU incorporation using the Click-iT™ EdU kit (Invitrogen), according to the manufacturer protocol. In brief, 2 µM of EdU labeling solution was added to the cultures and incubated for 2 days before the cultures were fixed with 4% paraformaldehyde (PFA) (Electron Microscopy Sciences, Hatfield, PA, USA) for 15 min. After washing and permeabilization with 0.5% Triton^®^ X-100 (Sigma-Aldrich) for 20 min, the Click-iT^®^ reaction cocktail was added, and after 30 min the proliferating cells were identified by imaging using Olympus IX81 microscope equipped with a 4x objective and a 488/527 filter.

### Immunofluorescent staining

Cultures were fixed with 4% PFA for 15 min followed by 1 h blocking in 10% horse serum (Gibco, Grand Island, NY, USA) in IF buffer (PBS with 0.1% BSA, 0.2% Triton X-100 and 0.05% Tween-20 (Merck, Darmstadt, Germany)). The samples were incubated with primary antibodies (diluted in the IF-buffer as specified in the **Supplementary Table S2**) overnight at 4°C. After washing with IF buffer 3x10 min, the samples were incubated with secondary antibody and DAPI (as specified in **Supplementary Table S2**) in IF buffer for 2 h at room temperature, followed by washing with PBS 4x10 min. Imaging was performed using a Zeiss LSM 710 laser-scanning confocal microscope equipped with a Zeiss plan-Apochromat 20x NA/0.8 air objective (Carl Zeiss, Jena, Germany).

### Assessment of multidrug transporter functionality *ex vivo*

The fragments cultured in suspension for one week were collected and incubated with/without 10 μM verapamil (Sigma-Aldrich), an inhibitor of a multidrug transporter, for 30 min followed by incubation with 1 μM doxorubicin (Pfizer, New York, NY, USA) for 24 h at 37°C. The accumulation of doxorubicin in the fragments was analyzed by Olympus IX81 microscope equipped with a 10x objective and a 540/590 filter, and quantification was performed using an Olympus software Cell P, which separately measures mean color intensity per fragment within the image.

### Quantification of human/mouse DNA content and *ABCB1* mRNA level by real-time qPCR

Up to 30 mg of fresh frozen tumor tissue or 1x10^7^ cells were lysed in 600 µl RLT Plus buffer w/2 mM DTT using the QIAshredder homogenizer (Qiagen, Hilden, Germany). The instrument was operated for 2x4 min at a frequency of 30Hz. Homogenized lysate was passed through a QIAshredder spin column at 20000g for 30 s to remove debris. Genomic DNA and total RNA were simultaneously extracted from the lysates using the QIAcube instrument and the AllPrep DNA/RNA/miRNA Universal kit (all Qiagen).

To estimate the content of human and mouse DNA in each sample, we use the assay described previously (22). It is based on real-time qPCR using species-specific TaqMan probes conjugated with different fluorescent tags (human: tgctgcttctcattgtctcg (FAM) and mouse: cctgctgcttatcgtggctg (VIC)) along with common human/mouse forward (tacctgcagctgtacgccac) and reverse (gaccacctcattctcctggc) primers. The primer/probes detect the prostaglandin E receptor 2 (*PTGER2*) gene region, which is highly homologous between the two species and known not to be duplicated/deleted in disease. The standard curve (Ct values as a function of known amount of human and mouse DNA) were generated employing serially diluted DNA isolated from the human melanoma cell line WM115 and the mouse colon carcinoma cell line CT26. Real-time PCR was carried out on an BioRad CFX connect Real time System (Bio-Rad, Hercules, CA, USA) using 50 ng of total genomic DNA in 25 µl reaction mix containing 200 nM of each primer/probe (Applied Biosystems, Waltham, MA, USA) and 1x PerfeCTa qPCR ToughMix (Quanta Biosciences, Gaithersburg, MD, USA). The qPCR conditions were as follows: 5 min 95°C initial denaturation, 40 cycles of 15 s denaturation at 95°C and 30 s annealing/extension at 60°C. Quantifications were performed taking into account that one haploid mouse genome is approximately 2.9 pg, whereas one human haploid genome is approximately 3.33 pg. “Percent human (or mouse) DNA” was estimated as follows: [number of human (or mouse) genome]*100/[sum human+mouse genome].

To detect *ABCB1* gene mRNA level, extracted total RNA was converted into cDNA using the qScript cDNA Synthesis Kit (Quanta Biosciences). PCR was carried out as specified above using 50 ng cDNA, *ABCB1* forward (5’gaaatttagaagatctgatgtcaaaca’3) and reverse (5’actgtaataataggcatacctggtca’3) primers (Integrated DNA Technologies, Leuven, Belgium) and 10 µM probe #65 from Universal Probe Library (Roche Applied Science, Penzberg, Germany). The reference gene *TBP* was detected using the commercially available Applied BioSystems TaqMan Assay. Relative gene expression was calculated using the ΔΔ Ct method.

## Results

### Isogenic PDXs with distinct sensitivity to paclitaxel

To establish *ex vivo* models that recapitulate treatment responses seen in individual tumors, we have utilized the previously described TNBC PDX, MAS98.12 (18) and its chemoresistant derivative, MAS98.12PR. *In vivo*, MAS98.12 was highly sensitive to paclitaxel (**Figure 1**). One of the regressed tumors, however, started to re-grow after ten weeks (**Figure 1A**, dashed line) and was unresponsive to repeated treatment with paclitaxel. This tumor was the origin of the paclitaxel-resistant sub-line, MAS98.12PR. Later generations of MAS98.12PR tumors retained resistance to paclitaxel (**Figure 1B**). This pair of isogenic PDXs has been used as a source of human tumor tissue that originates from the same patient but differs with respect to paclitaxel sensitivity. We aimed to establish tissue cultures that recapitulate this difference *ex vivo*.

### *Ex vivo* proliferative capacity of the dissociated PDX tissue

**Figure 2:**
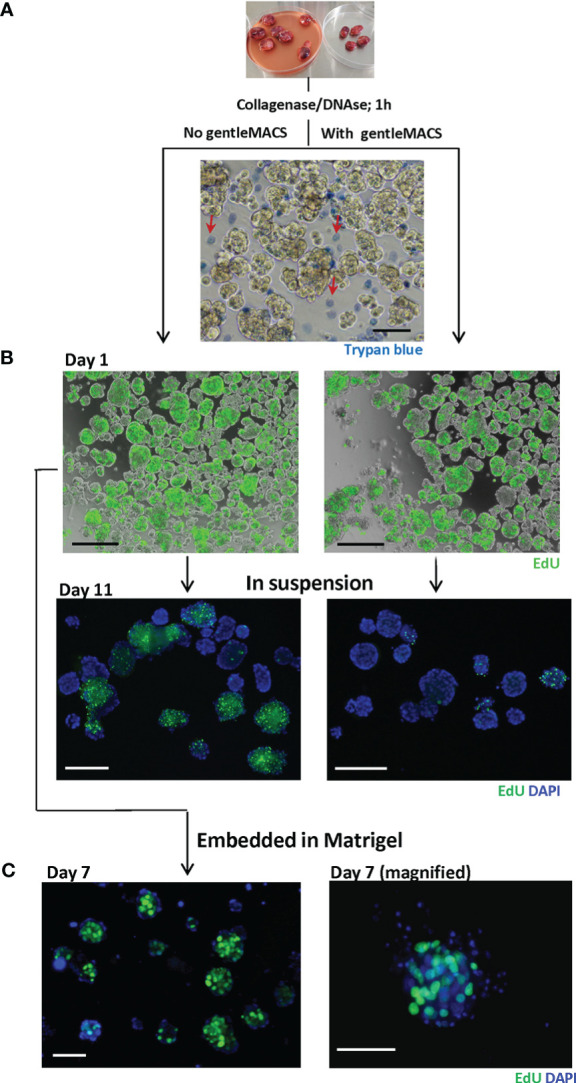
Dissociated MAS98.12/MAS98.12PR PDX tissue; appearance and proliferative capacity *ex vivo*. Tumors were disintegrated using collagenase/DNAse with/without gentleMACS. The resulting tissue suspension was stained with trypan blue **(A)** to identify dead cells and EdU **(B)** to evaluate the proliferating capacity. The EdU staining was also performed on 7/11 day-cultures either in suspension **(B)** or in Matrigel **(C)**; DAPI stains the nucleus; scale bars, 100 μm.

To dissociate BC tissue for subsequent culturing, we tested two methods used in similar studies: the conventional enzymatic digestion using collagenase IV/DNAse for up to 1 h (11–13), and the additional mechanical homogenization using gentleMACS dissociator, as used by Guillen et al. (10). Regardless of the dissociation method and the PDX model, the resulting tissue suspension consisted of single cells and small non-disrupted tissue fragments as shown in **Figure 2**. Trypan blue staining revealed lower viability among single cells than fragments, which mostly harbored trypan blue-negative viable cells (**Figure 2A**). GentleMACS increased the recovery of single cells (data not shown), though the non-disrupted tissue fragments were still present at significant amounts. To note, enzymatic digestion overnight disrupted the fragments to single cells, but cell viability was very low (data not shown).

The proliferative capacity of the cells isolated by the two methods was further compared by measuring incorporation of EdU. The dissociated tissue was cultured in suspension and, at different time points, stained with EdU. On day 1, no obvious difference with respect to EdU-incorporation was observed between the two methods, indicating a similar proliferative capacity of the dissociated tissue. With time, however, cultures prepared with gentleMACS lost their proliferative capacity, while the cultures processed by the enzymatic digestion only, kept proliferating for at least 11 days (**Figure 2B**). The proliferative capacity was also maintained when the dissociated tissue was cultured within Matrigel (**Figure 2C**). In both suspension and Matrigel cultures, the EdU positive cells were primarily found in the tissue fragments, although there was substantial heterogeneity between and within the fragments (**Figures 2B, C**).

### The tissue fragments harbor human tumor cells

**Figure 3:**
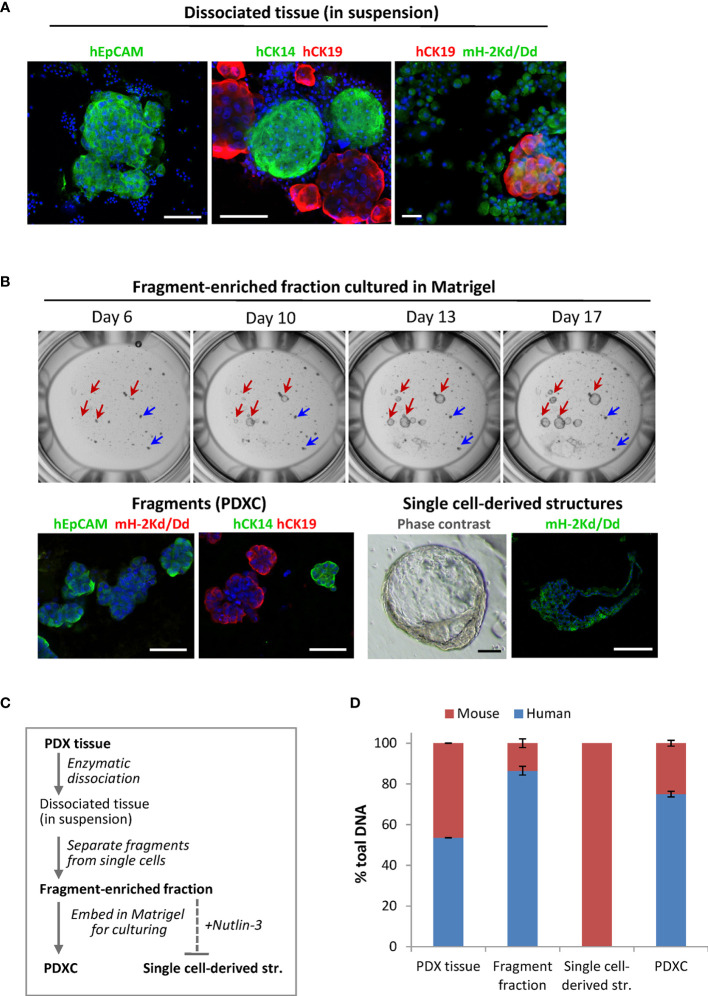
The composition of the dissociated MAS98.12/MAS98.12PR PDXs and the *ex vivo* cultures. **(A)** IF staining of the dissociated PDX tissue suspension with human EpCAM, CK14 and CK19, and mouse H-2Kd/Dd; scale bars, 100 μm. **(B)** Cultures established from fragment-enriched fraction embedded in Matrigel. Upper panel: phase contrast pictures taken over time, where fragments and single cell-derived structures are shown by blue and red arrows, respectively. Lower panel: IF staining with human EpCAM, CK14 and CK19 and mouse H-2Kd of fragments and single cell-derived structures; DAPI stains the nucleus; scale bars, 100 μm. **(C)** The scheme indicating preparation of the samples discussed in the figure; bold indicates the samples whose DNA composition was analyzed by species-specific qPCR and presented in **(D)**; **(D)** Quantitative assessment of human and mouse DNA content in the different samples; average ± StDv (n=2, here represented by one sample from each PDX).

To analyze the composition of the dissociated PDX tissue, we performed immunostaining using species-specific antibodies. To identify mammary cells of human origin, we stained for the human epithelial markers: epithelial cell adhesion molecule EpCAM, myoepithelial/basal cytokeratin CK14 and luminal cytokeratin CK19. In both PDX models, the fragments were positive for these markers, while the single cells outside the fragments were negative, suggesting their mouse origin (**Figure 3A**). Staining with the mouse-specific MHC class-I molecule H-2Kd/Dd validated that majority of the cells outside the fragments are H-2Kd/Dd-positive mouse cells (**Figure 3A**). Based on this data, we introduced additional steps (multiple low-speed centrifugations and size-based separation as specified in Materials and Methods) to separate the fragments. The fragment-enriched fraction was embedded in Matrigel for further culturing as explants *ex vivo*. In such cultures, the fragment size did not change significantly over time (**Figure 3B** upper panel, blue arrows). However, we observed single cell-derived structures that increased notably in diameter during culturing (**Figure 3B** upper panel, red arrows), similar to what has been reported for normal- or BC-organoids (11, 12). Immunostaining with human EpCAM, CK14, CK19 and mouse H-2Kd/Dd validated that the fragments consisted of human epithelial tumor cells. In contrast, the singe-cell derived structures consisted of mouse cells surrounding the cavity (**Figure 3B**, lower panel), suggesting that they were organoids of mouse origin. To eliminate mouse cells forming such structures, we applied Nutlin-3 selection. As shown previously, treatment with Nutlin-3 eliminates normal organoids, but does not affect cancer organoids with *TP53* mutation (11), and MAS98.12 harbors mutation in the *TP53* gene (18). In Nutlin-3 pre-treated explants, we observed reduced amount of mouse cells and the absence of mouse organoids. Such explants are further called PDXCs.

To further validate the origin of the different samples along the PDXCs preparation (specified in **Figure 3C**), we quantified human and mouse DNA content by qPCR using species-specific probes for the *PTGER2* gene. As expected, in single-cell derived structures only mouse *PTGER2* was detected (Ct values around 26) (**Figure 3D**). In the isolated fragment-enriched fraction and the eventual PDXCs, human DNA was clearly dominant, though some contamination with mouse DNA should be noted (**Figure 3D**). For comparison, the original PDX tissue contained approximate equal amounts of human and mouse DNA. Altogether, this data indicates that fragments are the main source of human tumor cells in the dissociated tissue from the investigated PDXs.

### The resistant PDXs and PDXCs over-express ABCB1 transporter that is functional *ex vivo*

**Figure 4:**
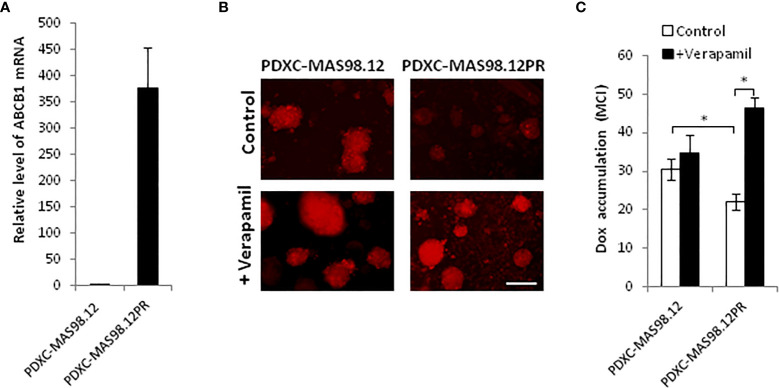
MAS98.12PR-derived cultures over-express ABCB1 that is functionally active *ex vivo*. **(A)** Relative expression of *ABCB1* gene in 10 d-cultures from MAS98.12PR compared to MAS98.12 (set to 1); average ± SEM (n=4 (2 for suspension and 2 for Matrigel cultures)). **(B, C)** Accumulation of doxorubicin (Dox) in 10d-cultures from MAS98.12 and MAS98.12PR after 24h-incubation with 1 µM Dox in the presence or absence of 10 µM verapamil. Representative fluorescence pictures **(B)** and quantified Dox accumulation presented as mean color intensity (MCI) in the fragments (average ± SEM (n≥7) **(C)**; scale bar, 200 μm; *, p < 0.05 by unpaired t-test. **(C)**.

To investigate whether PDXCs from MAS98.12 and MAS98.12PR retain the molecular properties of the parental PDXs, we measured the expression of the characteristic genes. Previously performed gene expression profiling of this PDX pair identified A*BCB1* (*MDR1*), which encodes a multidrug ABC transporter, as the most differentially expressed gene, with approximately 16-fold up-regulation in MAS98.12PR compared to MAS98.12 (data not shown). Correspondingly, we detected a significantly higher level of *ABCB1* mRNA in the cultures from MAS98.12PR compared to MAS98.12 cultures (**Figure 4A**).

Up-regulation of ABC transporters is a well-described mechanism of chemoresistance that reduces cellular accumulation of drugs (23). To investigate whether the over-expressed transporter encoded by *ABCB1* was functional *ex vivo*, we analyzed cellular accumulation of a fluorescent drug doxorubicin. Cultures from MAS98.12PR accumulated notably less doxorubicin compared to the cultures from MAS98.12 (**Figures 4B, C**). In the presence of verapamil, the inhibitor of ABC transporters, the accumulation of doxorubicin was increased in the cultures from MAS98.12PR and reached the same levels as in the MAS98.12 cultures (**Figures 4B, C**). Altogether, this indicates that *ABCB1* expression difference seen in the PDXs is retained in the respective cultures, and that the transporter is active *ex vivo*.

### PDXCs recapitulate paclitaxel-resistance of the PDXs

**Figure 5:**
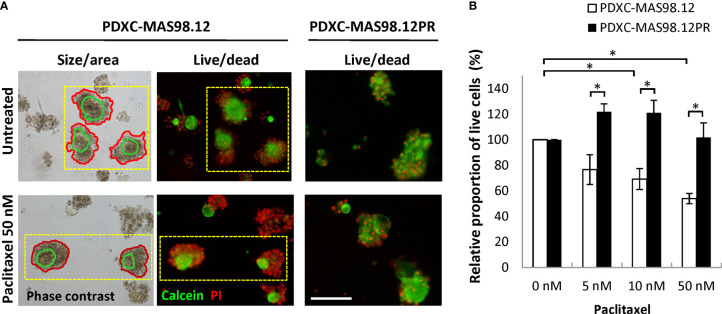
Sensitivity of MAS98.12- and MAS98.12PR-derived PDXCs to paclitaxel. Untreated and paclitaxel treated for one week PDXCs in Matrigel were stained with calcein/PI and a proportion of viable cells was quantified. **(A)** Representative pictures, where the red lines in the phase contrast pictures (left) mark the automatically detected fragment area, and the green line marks the “live” part, as validated by the fluorescence pictures (middle); scale bar, 200 μm. **(B)** A proportion of viable cells in the treated cultures presented as a percentage of the respective untreated controls; average ± SEM (n=4; where either fresh (n=2) or cryopreserved (n=2) PDX tissue was used to establish PDXCs, see **Supplementary Figure S1**); *, p < 0.05 by unpaired t-test.

To assess whether the difference in paclitaxel sensitivity seen in the PDXs (**Figure 1B**) can be recapitulated in the respective PDXCs, we treated the explants with increasing concentrations of paclitaxel for one week. The treatment efficacy was evaluated by three different read-out strategies. First, we attempted to automatically monitor fragment size/total area by the Incucyte equipped with the organoid module. Unfortunately, this approach was unsuitable for the cultures from the MAS98.12/MAS98.12PR PDXs (to note, it was useful for cultures from other PDXs. such as HBCx39 as shown below. After treatment, high numbers of dead cells were found in the periphery of the fragments; this contributed to the fragment size, impairing correlation between size and viability/growth (**Figure 5A**, left panel). Therefore, we employed another read-out based on live and dead staining with calcein and PI, respectively (**Figure 5A** middle/right panel), followed by microscopy-based quantification of the proportion of live cells in the fragments. As shown in **Figure 5B**, the PDXCs from MAS98.12 demonstrated a paclitaxel dose-dependent decrease in the proportion of viable cells. In concordance, the extent of dead, PI-positive cells was increased as illustrated in **Figure 5A**. On the contrary, the PDXCs from MAS98.12PR showed no decrease in the proportion of viable cells (**Figure 5B**) and no increased staining with PI (**Figure 5A**), indicating their insensitivity to paclitaxel. Importantly, similar differences in sensitivity were observed in both cultures from fresh and cryopreserved tumor tissue (**Supplementary Figure S1**). Finally, we applied the conventional CTG assay that measures bulk metabolic activity in the cultures. The results matched the live-dead staining (except at the highest dose of paclitaxel), revealing the paclitaxel resistance in cultures from MAS98.12PR (**Supplementary Figure S2A**). However, we noted big variation between parallel wells. Furthermore, it was not possible to discriminate the impact from the contaminating mouse cells, which could explain equal drop in metabolic activity in both PDXCs at the highest dose of paclitaxel.

**Figure 6:**
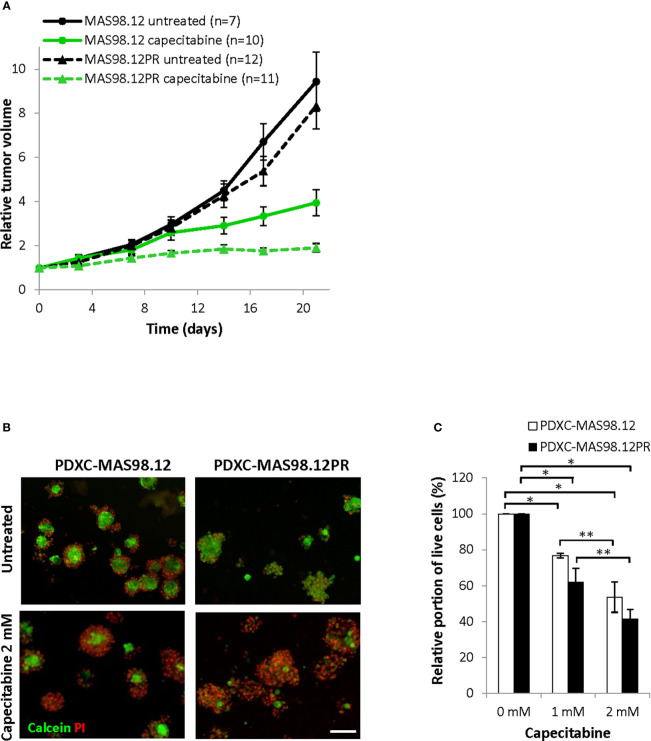
Sensitivity of MAS98.12/MAS98.12PR PDXs and the respective PDXCs to capecitabine. **(A)** Relative tumor volume (normalized to the volume at the day when the treatment was started) of non-treated and capecitabine-treated (540 mg/kg, 5x/week) MAS98.12 and MAS98.12PR PDX; average ± SEM (n indicated in the legend). **(B, C)** PDXCs in Matrigel with/without capecitabine treatment for one week followed by calcein/PI staining (representative pictures in **(B)**) to quantify the proportion of viable cells among all cells **(C)**. **(B)** Representative fluorescent pictures; scale bar, 200 μm. **(C)** A proportion of viable cells in the treated cultures presented as a percentage of the respective untreated controls; average ± SEM (n=3); * and **, p < 0.05 by unpaired and paired t-test, respectively.

Further, we compared the PDXCs sensitivity to another chemotherapeutic agent, capecitabine (a 5-FU pro-drug). This drug is commonly used as a salvage therapy in patients with remaining TNBC after pre-operative chemotherapy (24). *In vivo*, capecitabine treatment notably inhibited tumor growth in both MAS98.12 and MAS98.12PR, and the latter even showed a slightly better response (**Figure 6A**). In line with the *in vivo* data, both PDXCs showed good dose-dependent response to capecitabine as quantified by live-dead staining and the CTG assay (**Figures 6B, C** and **Supplementary Figure S2B**). Furthermore, PDXC-MAS98.12PR showed a tendency for a slightly better response than PDXC-MAS98.12 (**Figure 6C**).

### HBCx39-derived PDXCs recapitulate the drug sensitivity profile of the parental PDX

To investigate PDXCs from another patient, we employed HBCx39 PDX, which also represent TNBC. Similar to MAS98.12, the dissociated HBCx39 consisted of non-disrupted tissue fragments positive for human epithelial markers, EpCAM, CK14 and CK19 (**Supplementary Figure S3A**). The HBCx39-derived fragments were effectively growing *ex vivo* (**Supplementary Figure S3BA**). The explants showed high viability as revealed by calcein/PI staining (**Supplementary Figure S3BB**) and represented human tumor tissue as they stained with human-specific mitochondria and panCK antibodies and were mostly negative for mouse H-2Kd/Dd (**Supplementary Figure S3C**). Due to efficient growth, high viability and well-defined periphery of the fragments, PDXCs from HBCx39 could be easily analyzed by monitoring fragment size as a read-out of treatment efficacy.

**Figure 7:**
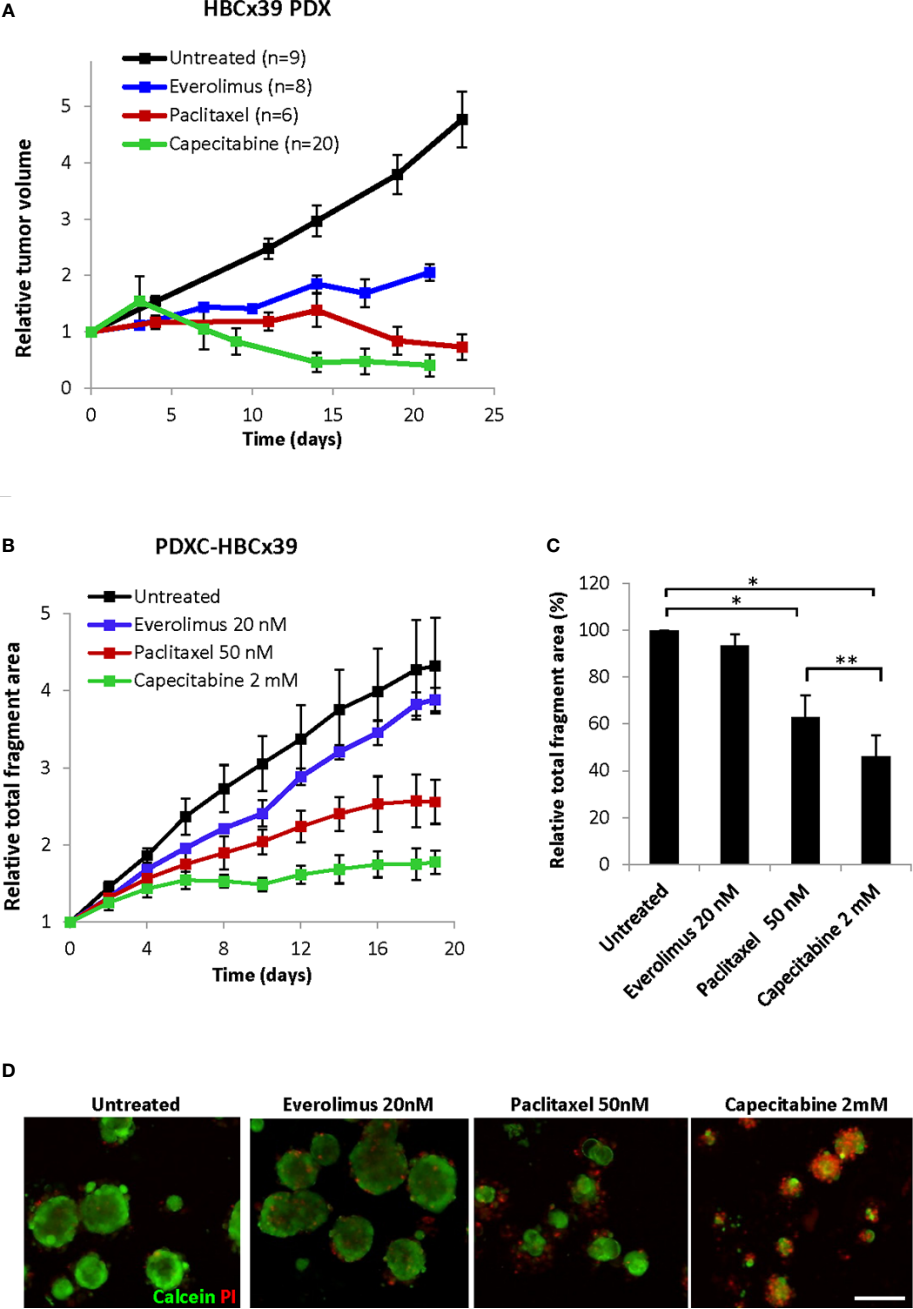
Response of HBCx39 PDX and PDXC to paclitaxel, capecitabine and everolimus. **(A)** Relative tumor volume (normalized to the volume at the day when the treatment was started) of non-treated and treated HBCx39 PDX; the treatment was as follows: paclitaxel (15 mg/kg, 2x/week), capecitabine (755 mg/kg, 5x/week) and everolimus (5 mg/kg, 5x/week). **(B)** Relative total fragment area normalized to the area at the start of the treatment; average ± StDv (3-4 parallels for each condition in one representative experiment). **(C)** The total fragment area in the treated cultures (day 19) shown as a percentage of the untreated controls; average ± SEM (n=3 for paclitaxel and capecitabine) and ± StDv (n=2 for everolimus); * and **, p < 0.05 by unpaired and paired t-test, respectively. **(D)** Representative fluorescence pictures of PDXCs treated with/without the indicated drugs for 19 days before staining with calcein and PI; scale bar, 200 μm.

*In vivo* HBCx39 PDXs showed high sensitivity to paclitaxel and particularly capecitabine, and lower sensitivity to the mTOR inhibitor everolimus (**Figure 7A** and **Supplementary Figure S4**). To investigate whether such sensitivity differences are recapitulated *ex vivo*, we treated HBCx39-derived PDXCs with these drugs and followed changes in fragment size over time by the Incucyte organoid module. As expected, a time-dependent increase in the total fragment area was detected in the untreated controls (**Figure 7B**). Everolimus (20 nM) induced a low growth inhibitory effect, while paclitaxel significantly reduced and capecitabine completely abrogated the fragment growth (**Figures 7B, C** and **Supplementary Figures S5A, B**). Similar difference in sensitivity was registered also by the CTG assay (**Supplementary Figure S5C**) and further validated by live/dead staining (**Figure 7D**). The latter also revealed substantial cell death at day 19 upon capecitabine treatment, while the effect of paclitaxel was strongly cytostatic with much fewer dead cells observed (**Figure 7D**). These observations correlated nicely to the *in vivo* results of long follow-up, where on week 7 we observed complete regression of all tumors in the capecitabine group, while the paclitaxel group carried small residual tumors (**Supplementary Figure S4**).

## Discussion

In this study, we have recapitulated *in vivo* drug responses using patient-derived tissue cultures from BC. With the applied “know-how” reported in the previous publications (10–13), short-term explants from PDXs with distinct drug sensitivity were established, and possibilities to detect concordant responses were validated. BC PDOs and PDEs are being considered as attractive tools for predicting drug sensitivity in individual tumors, though their application has not been straightforward. Although we succeeded in demonstrating matching differences in drug responsiveness between PDXs and PDXCs, several challenges were encountered. First, conventional enzymatic dissociation of BC tissue resulted in a mix of single cells and difficult-to-disrupt tissue fragments. Many applications, including generation of organoids, single-cell RNAseq or cytometric analyses, require single cells. However, in the dissociated PDX tissue, the single cells showed low viability. Furthermore, they represented mostly normal host cells, including mammary progenitors able to generate organoid-like structures of mouse origin. Viable human tumor cells were retained within the non-disrupted tissue fragments, indicating the possibility to maintain tumor tissue *ex vivo* by culturing such fragments as explants. The “behavior” of the fragments in culture, however, depended on the characteristics of the PDX tissue. In this study, one of the aims was to recapitulate sensitivity and resistance to paclitaxel as found in the isogenic pair of PDXs, MAS98.12 and MAS9812PR. However, tissue from these PDXs appeared to be challenging to culture. Although we confirmed the presence of viable and proliferating tumor cells, the MAS98.12/MAS98.12PR-derived fragments demonstrated limited growth, in contrast to the fragments from the other PDX, HBCx39. Furthermore, a notable number of dead cells were associated with the cultured fragments from MAS98.12/MAS98.12PR, which was not the case for HBCx39 cultures. Those features influenced the choice of a method for monitoring treatment efficacy. Tracking changes in fragment size was a suitable and easy read-out for cultures from HBCx39, but not MAS98.12/MAS98.12PR. For cultures from MAS98.12/MAS98.12PR, estimation of the proportion of live cells based on imaging was a suitable approach, allowing to capture the cytotoxic influence of the drug. Since this method estimates the ratio between two signals (live and dead) and not a total signal in a well (like e.g. CTG), it is not obstructed by the different amount/size of the fragments in individual wells. The latter has been difficult to avoid due to heterogeneity of the dissociated tissue, which affected the CTG measurements, where we noted big variations between parallels. Furthermore, the CTG assay gives no possibility to discriminate the fragment-signal from the signal of the “contaminating” normal cells, which was possible by the imaging-based approaches. Despite those limitations, the CTG assay generally recapitulated the effects registered by other methods, where the mentioned concerns could be controlled. In conclusion, the choice of an optimal read-out method might depend on the cultured tissue characteristics and might require individual adjustment.

Despite those technical challenges, we succeeded in recapitulating paclitaxel-resistance and -sensitivity in the PDXCs from the resistant and the sensitive PDXs, respectively. This has been demonstrated in cultures from both fresh and cryopreserved tissue, and the latter might be advantageous when collecting tissue directly from a patient. The difference in response was not an artifact associated with the tissue, but was drug-dependent i.e. observed for paclitaxel, but not capecitabine, as in the matching PDXs. Furthermore, we recapitulated drug sensitivity profile of another PDX, HBCx39, where superior responses to capecitabine, compared to paclitaxel or everolimus, were observed also in the PDXCs. Finally, we addressed a common technical challenge of the PDX cultures *i.e.* contamination with normal host cells, which could be reduced by the treatment with the MDM2 inhibitor Nutlin-3. This, however, is a suitable approach only for tumors with lost/mutated *TP53* (25), which is commonly observed among TNBC. For tumors with wild-type *TP53*, alternative approaches are needed, and one of such is separation based on physical parameters, like size. We have employed size-based filtering combined with low-speed centrifugation and thereby facilitated separation of tumor fragments from contaminating normal single cells.

Taken together, the presented data demonstrates the feasibility of employing tissue explants to “capture” drug sensitivity of individual tumors, which supports the predictive potential of the *ex vivo* platform. The established protocols will facilitate setting up an analogous platform for BC patient biopsies with the aim to facilitate functional precision medicine. It would be highly useful to determine *ex vivo* the sensitivity to e.g. salvage therapy as recommended for TNBC and HER2^+^ patients that have shown less-than-optimal response to neo-adjuvant chemotherapy (24, 26).

## Data availability statement

The raw data supporting the conclusions of this article will be made available by the authors, without undue reservation.

## Ethics statement

The animal study was reviewed and approved by the Norwegian Food Safety Authority (FOTS id 15499).

## Author contributions

SP performed all *ex vivo* experiments and immunostaining, data analysis and interpretation, and contributed to making the respective figures and writing the manuscript; GØ performed all *in vivo* work and PCR analysis; EE performed gene expression profiling of the PDXs and contributed to the revision of the manuscript; SJ contributed to establishment of tissue dissociation conditions; OE and GM participated in study design, result interpretation, drawing the conclusions, and contributed to writing the manuscript; LP designed the study, contributed to the data analysis, result interpretation, preparation of the figures, and wrote the manuscript. All authors critically evaluated the results and reviewed the manuscript. All authors contributed to the article and approved the submitted version.
