# Supplementary material for: Breast cancer patient-derived explant cultures recapitulate in vivo drug responses
Source: Front Oncol. 2023 Feb 22;13:1040665. doi: 10.3389/fonc.2023.1040665 (PMC9992973; doi:10.3389/fonc.2023.1040665)
Supplement: Supplementary file 1 [file DataSheet_1.pdf]

Supplementary Table S1. Organoid Medium (OM and OM+*) composition.

OM components	Supplier	Catalogue #	Final concentration
Advanced DMEM/F12	Gibco	12634010	1x
Penicillin/Streptomycin	Gibco	15140-122	100 U/ml
GlutaMax 100x	Gibco	35050061	1x
Hepes	Gibco	15630056	10 mM
R-Spondin 3	Peprotech	120-44	250 ng/ml
Neuregulin 1	Peprotech	100-03	5 nM
FGF 7	Peprotech	100-19	5 ng/ml
FGF 10	Peprotech	100-26	20 ng/ml
EGF	Peprotech	AF-100-15	5 ng/ml
Noggin	Peprotech	120-10C	100 ng/ml
A83-01	Tocris	2939	500 nM
Y-27632	Abmole	Y-27632	10 μM
SB202190	Sigma	S7067	500 nM
B27 supplement	Gibco	17504-44	1x
N-Acetylcysteine	Sigma	A9165-5g	1.25 mM
Nicotinamide	Sigma	N0636	5 mM

*OM+, as OM with added 5 μM Nutlin-3 (Cayman, Cat. # 10004372).

Supplementary Table S2. Antibodies/dyes used for immunofluorescent staining.

Primary antibodies/dyes	Supplier	Catalogue #	Dilution
human EpCAM	BioLegend	324202	100
human CK14	Novocastra	NCL-L-LL002	100
human CK19	Novus Bio	NBP1-78278	100
human Mitochondria	AbCam	ab92824	800
human PanCK	AbCam	ab86734	100
mouse H-2Kd/Dd	Invitrogen	MA5-18004	600
Alexa 546 Phalloidin (F-actin)	Molecular Probes	A22283	50
DAPI	Sigma-Aldrich	D9542	1 μg/ml
**Secondary antibodies**			
goat anti-mouse Alexa 488	Invitrogen	21141	800
donkey anti-mouse Alexa 488	Jackson ImmunoResearch	715-545-150	400
donkey anti-rabbit Alexa 555	Invitrogen	A32794	200
goat anti-mouse Alexa 568	Invitrogen	A21043	800

Supplementary Figure S1

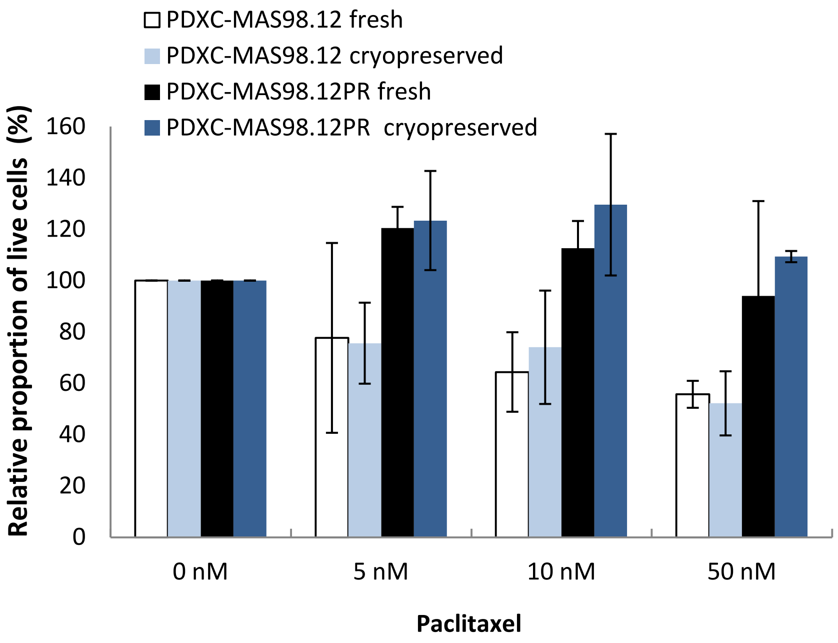

Supplementary Figure S1. Response to paclitaxel in PDXCs from fresh *versus* cryopreserved MAS98.12 and MAS98.12PR PDXs tissue. PDXCs in Matrigel prepared from either fresh of cryopreserved tumor tissue were treated with paclitaxel for one week before staining with calcein/PI to quantify a proportion of viable cells in each condition. The data are presented as a percentage of the respective untreated controls; average ± StDv (n=2). The merged data (from fresh and cryopreserved tissue) are shown in Figure 5.

Supplementary Figure S2

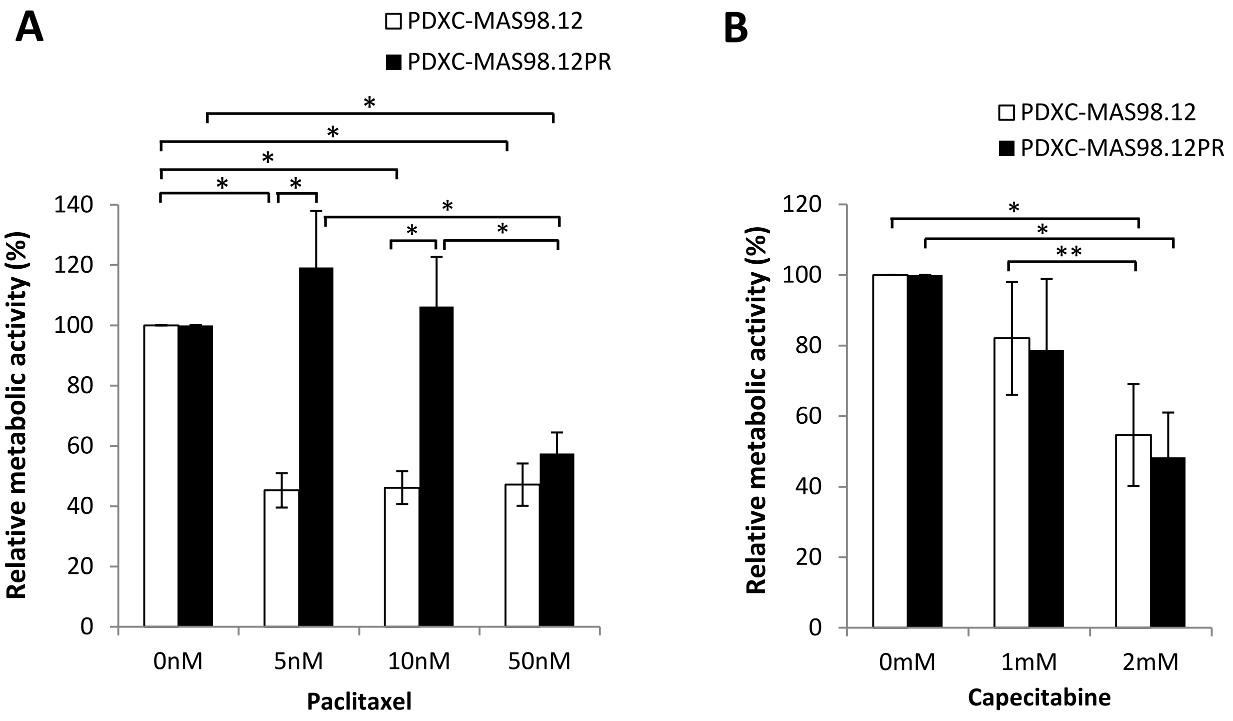

Supplementary Figure S2. MAS98.12/MAS98.12PR-derived PDXCs response to paclitaxel and capecitabine as scored by measuring metabolic activity. The PDXCs in Matrigel were treated with the indicated concentrations of paclitaxel (A) or capecitabine (B) for one week before measurement of the treatment effect by the CTG assay; the values in treated cultures are presented as a percentage of the respective untreated controls; average ± SEM (n=3); * and **, $p < 0.05$ by unpaired and paired t-test, respectively.

Supplementary Figure S3

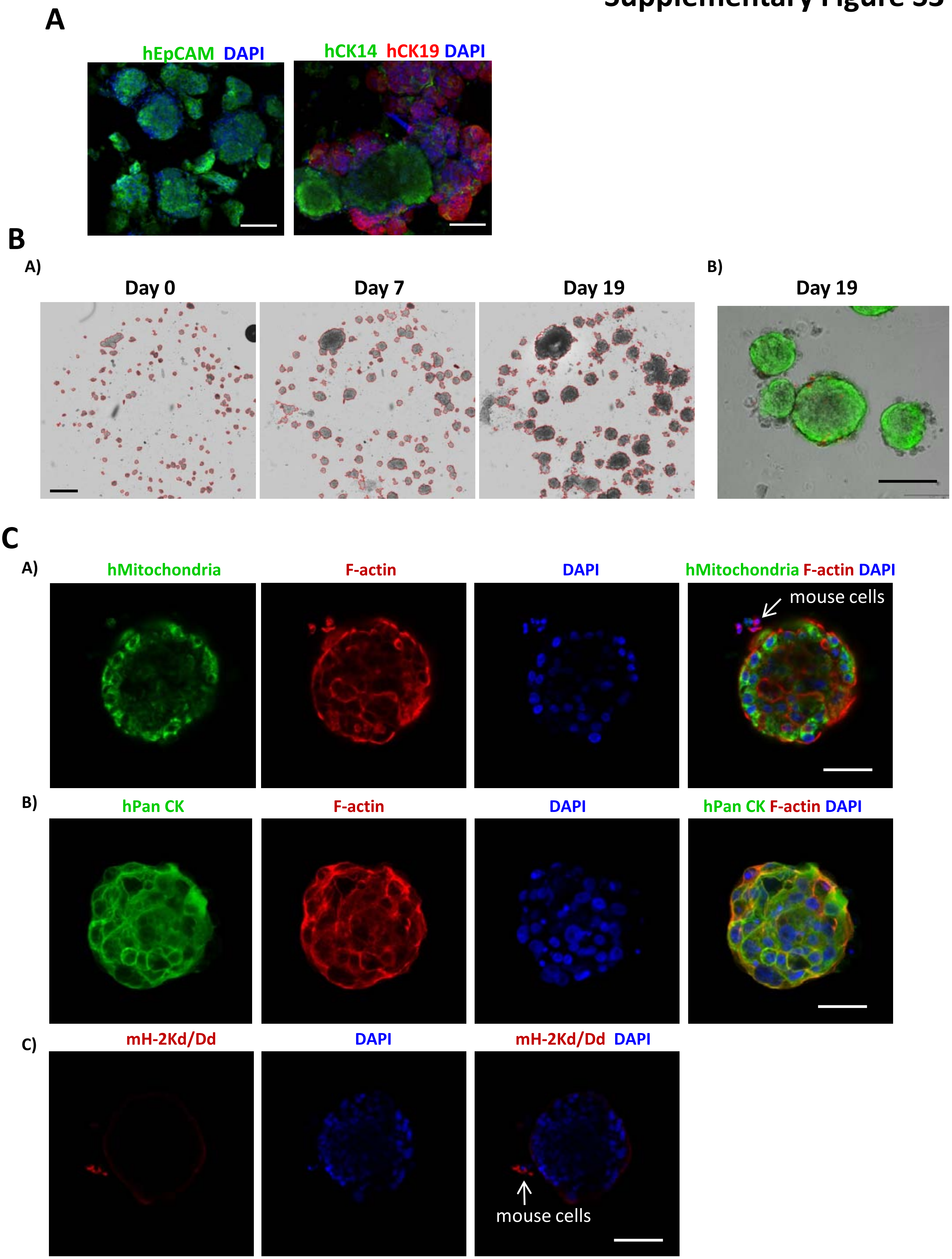

Supplementary Figure S3. Characterization of HBCx39-derived PDXCs. (A) IF staining of the dissociated HBCx39 PDX tissue suspension with human EpCAM, CK14 and CK19 (DAPI stains the nucleus); scale bars, 100 µm. (B) Growth of fragments isolated from HBCx39 and embedded in Matrigel; phase contrast pictures taken over time (A; scale bar 500 µm) and calcein/PI staining pictures of 19d-cultures (B; scale bar 200 µm). (C) IF staining of the 19 d-cultures stained with human mitochondria (A), human panCK (B) and mouse H-2Kd/Dd (C) (cytoskeleton (F-actin) and nucleus (DAPI) stainings are shown); single mouse cells found in the cultures are indicated by arrows; scale bars, 100 µm.

Supplementary Figure S4

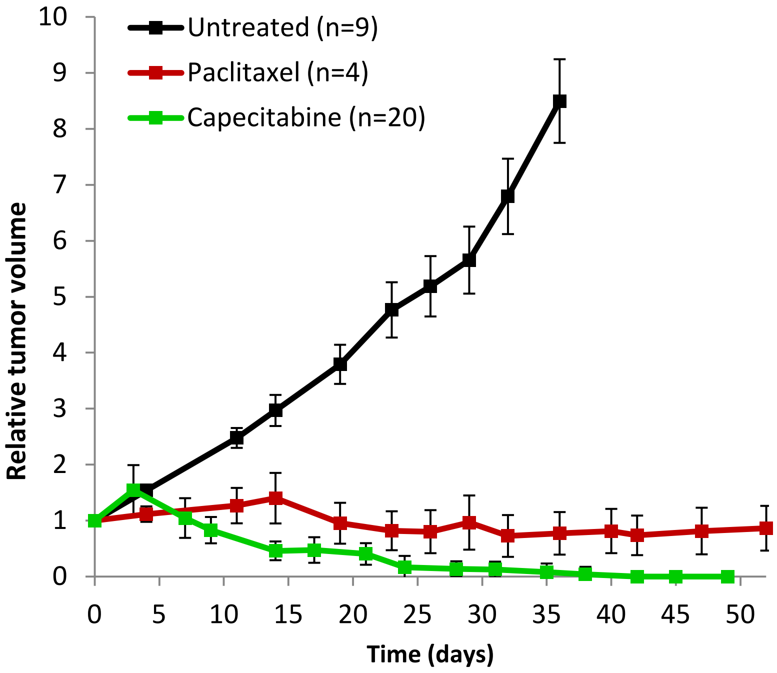

Supplementary Figure S4. Response of HBCx39 PDX to paclitaxel and capecitabine; prolonged follow-up of the treated tumors from Figure 7. The treatment was performed as specified in Fig. 7 (i.e. paclitaxel (15 mg/kg, 2x/week) and capecitabine (755 mg/kg, 5x/week). Relative volume of the treated tumors that were followed for 7 weeks is shown (average ± SEM, n indicated in the legend).

Supplementary Figure S5

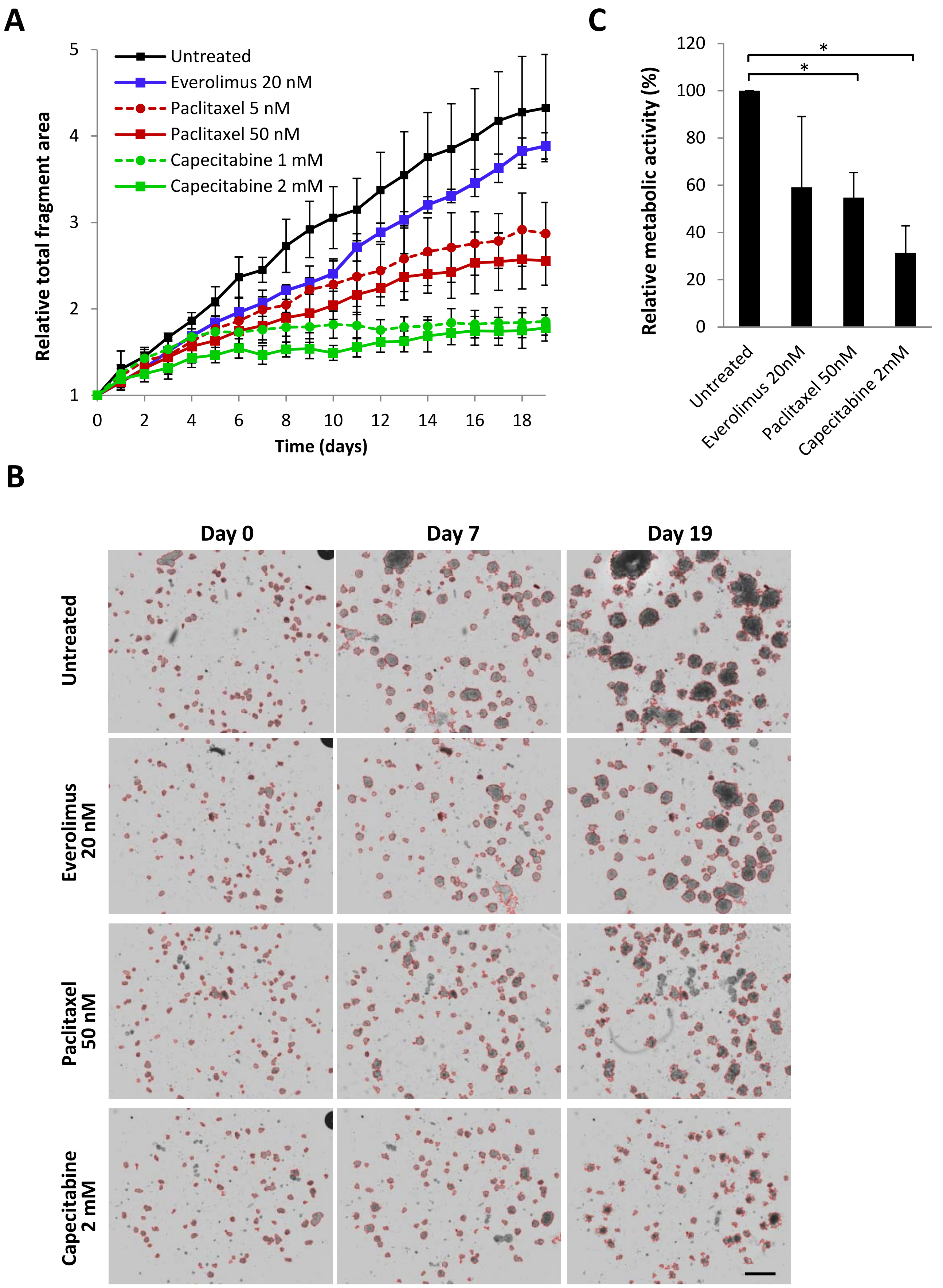

Supplementary Figure S5. Response of HBCx39-derived PDXCs to paclitaxel, capecitabine and everolimus. A) Relative fragment area normalized to the area at the start of the treatment; average ± StDv ($n \geq 10$ in one representative experiment); extended Fig. 7C, showing the effect of additional doses of paclitaxel (5 nM) and capecitabine (1 mM). B) Phase contrast pictures of the treated and control PDXCs over time. Red lines indicate the fragment borders detected by the Incucyte S3 organoid analysis software module and used to calculate the total fragment area; scale bar 500 μm. C) Response to the indicated drugs as measured by the CTG assay detecting the metabolic activity; the values in treated PDXCs are presented in percentage of the respective untreated controls; average ± SEM (n=3 for paclitaxel and capecitabine) and ± StDv (n=2 for everolimus); *, $p < 0.05$ by unpaired t-test.
